# Vaccination coverage among older adults: a population-based study in India

**DOI:** 10.2471/BLT.21.287390

**Published:** 2022-04-26

**Authors:** Ali Abbas Rizvi, Abhishek Singh

**Affiliations:** a International Institute for Population Sciences, Govandi Station Road, Deonar, Mumbai, Maharashtra, 400088, India.

## Abstract

**Objective:**

To estimate the prevalence and explore the predictors of vaccine uptake among older adults in India.

**Methods:**

We used data from the national Longitudinal Ageing Study in India, a national household survey conducted during 2017–2018. Based on interviewees’ self-reports, we calculated population-weighted estimates of the uptake of influenza, pneumococcal, typhoid and hepatitis B vaccines among 64 714 Indian adults aged 45 years or older. We performed multivariable binary logistic regression analysis to examine the sociodemographic and health-related predictors of uptake of the vaccinations.

**Findings:**

The coverage of each of the studied vaccinations was less than 2%. The estimated percentages of respondents reporting ever being vaccinated were 1.5% (95% confidence interval, CI: 1.4–1.6) for influenza, 0.6% (95% CI: 0.6–0.7) for pneumococcal disease, 1.9% (95% CI: 1.8–2.0) for typhoid and 1.9% (95% CI: 1.8–2.0) for hepatitis B. Vaccine uptake was higher among respondents with cardiovascular disease, diabetes or lung disease than those without any of these conditions. Uptake of influenza vaccine was higher among those with lung disease, while hepatitis B vaccine uptake was higher among those with cardiovascular disease or diabetes. Male sex, urban residence, wealthier household, more years of schooling, existing medical conditions and sedentary behaviours were significant predictors of vaccine uptake.

**Conclusion:**

Targeted policies and programmes are needed for improving the low vaccination coverage among older adults in India, especially among those with chronic diseases. Further research could examine vaccine access, vaccine hesitancy, and vaccine-related information and communication channels to older adults and their health-care providers.

## Résumé

**Objectif:**

Estimer la prévalence et explorer les facteurs prédictifs d’acceptation d’un vaccin chez les adultes plus âgés en Inde.

**Méthodes:**

Nous avons utilisé les données de l’Étude nationale longitudinale sur le vieillissement (Longitudinal Ageing Study) en Inde, enquête nationale auprès des ménages menée en 2017-2018. Sur la base des déclarations des personnes interrogées, nous avons calculé des estimations pondérées par la population de l’acceptation des vaccins contre la grippe, le pneumocoque, la typhoïde et l’hépatite B chez 64 714 adultes indiens âgés de 45 ans ou plus. Nous avons effectué une analyse de régression logistique binaire multivariable afin d’examiner les facteurs sociodémographiques et sanitaires liés à l’adoption des vaccins.

**Résultats:**

La couverture de chacune des vaccinations étudiées était inférieure à 2%. Les pourcentages estimés de répondants déclarant avoir déjà été vaccinés étaient de 1,5% (intervalle de confiance (IC) à 95% : 1,4-1,6) pour la grippe, de 0,6% (IC à 95% : 0,6-0,7) pour les infections à pneumocoques, de 1,9% (IC à 95% : 1,8-2,0) pour la typhoïde et de 1,9% (IC à 95% : 1,8-2,0) pour l’hépatite B. Le recours au vaccin était plus élevé chez les personnes interrogées souffrant de maladies cardiovasculaires, de diabète ou de maladies pulmonaires que chez celles ne présentant aucune de ces affections. Le taux d’acceptation du vaccin contre la grippe était plus élevé chez les personnes souffrant de maladies pulmonaires, tandis que le taux d’acceptation du vaccin contre l’hépatite B était plus élevé chez les personnes souffrant de maladies cardiovasculaires ou de diabète. Le sexe masculin, l’habitat en zone urbaine, les revenus plus élevés du ménage, le nombre d’années d’études, les problèmes de santé existants et les comportements sédentaires étaient des facteurs prédictifs significatifs d’acceptation du vaccin.

**Conclusion:**

Des politiques et des programmes ciblés sont nécessaires pour améliorer la faible couverture vaccinale chez les personnes plus âgées en Inde, en particulier chez celles atteintes de maladies chroniques. D’autres recherches pourraient examiner l’accès aux vaccins, l’hésitation à se faire vacciner, ainsi que les canaux d’information et de communication liés aux vaccins pour les adultes plus âgés et leurs prestataires de soins de santé.

## Resumen

**Objetivo:**

Estimar la prevalencia y explorar los predictores de la aceptación de la vacuna entre los adultos mayores en la India.

**Métodos:**

Se utilizaron datos del Estudio nacional de envejecimiento longitudinal en la India, una encuesta nacional de hogares realizada entre 2017 y 2018. A partir de los autoinformes de los entrevistados, se calcularon las estimaciones ponderadas a nivel poblacional de la aceptación de las vacunas antigripal, antineumocócica, antitifoidea y contra la hepatitis B entre 64 714 adultos indios de 45 años o más. Se realizó un análisis de regresión logística binaria multivariable para analizar los predictores sociodemográficos y sanitarios relacionados con la aceptación de las vacunas.

**Resultados:**

La cobertura de cada una de las vacunas estudiadas fue inferior al 2 %. Los porcentajes estimados de los encuestados que declararon haberse vacunado alguna vez fueron del 1,5 % (intervalo de confianza del 95 %, IC: 1,4-1,6) para la gripe, el 0,6 % (IC del 95 %: 0,6-0,7) para las enfermedades neumocócicas, el 1,9 % (IC del 95 %: 1,8-2,0) para la fiebre tifoidea y el 1,9 % (IC del 95 %: 1,8-2,0) para la hepatitis B. La aceptación de la vacuna fue mayor entre los encuestados con enfermedades cardiovasculares, diabetes o enfermedades pulmonares que entre los que no padecían ninguna de estas enfermedades. La aceptación de la vacuna antigripal fue mayor entre los que padecían enfermedades pulmonares, mientras que la aceptación de la vacuna contra la hepatitis B fue mayor entre los que padecían enfermedades cardiovasculares o diabetes. El sexo masculino, la residencia en una ciudad, un hogar más rico, un mayor número de años de escolarización, las enfermedades existentes y los comportamientos sedentarios fueron predictores significativos de la aceptación de la vacuna.

**Conclusión:**

Se necesitan políticas y programas específicos para mejorar la escasa cobertura de vacunación entre los adultos mayores de la India, en especial entre los que padecen enfermedades crónicas. Otras investigaciones podrían analizar el acceso a las vacunas, las dudas al respecto y los canales de información y comunicación relacionados con la vacunación para los adultos mayores y sus proveedores de atención médica.

## ملخص

**الغرض:**

لتقدير مدى الانتشار، واستكشاف عوامل التنبؤ بالحصول على اللقاح بين كبار السن في الهند.

**الطريقة:**

استخدمنا البيانات الواردة من دراسة الشيخوخة الطولية الوطنية في الهند، وهي مسح وطني للأسر تم إجراؤه خلال الفترة 2017 إلى 2018. بناءً على التقارير الذاتية للأشخاص الذين تمت مقابلتهم، قمنا بحساب التقديرات القائمة على السكان بخصوص الحصول على لقاحات الأنفلونزا، والمكورات الرئوية، والتيفوئيد والتهاب الكبد ب، بين 64714 بالغًا هنديًا تبلغ أعمارهم 45 عامًا أو أكبر. أجرينا تحليلًا للتحوّف اللوجستي الثنائي متعدد المتغيرات لفحص عوامل التنبؤ الاجتماعي السكاني والمتعلقة بالصحة والخاصة بالحصول على اللقاحات.

**النتائج:**

كانت تغطية كل من التطعيمات التي خضعت للدراسة أقل من 2%. كانت النسب المئوية التقديرية للمستجيبين الذين أبلغوا عن تلقيحهم في أي وقت مضى 1.5% (بفاصل ثقة مقداره 95%: 1.4 إلى 1.6) للأنفلونزا، و0.6 (بفاصل ثقة مقداره 95%: 0.6 إلى 0.7) لمرض المكورات الرئوية، و1.9% (بفاصل ثقة مقداره 95%: 1.8 إلى 2.0) لمرض التيفوئيد، و1.9% (بفاصل ثقة مقداره 95%: 1.8 إلى 2.0) لالتهاب الكبد ب. كان الحصول على اللقاح أعلى بين المستجيبين المصابين بأمراض القلب والأوعية الدموية، أو السكري، أو أمراض الرئة من أولئك الذين لا يعانون من أي من هذه الأمراض. كان الحصول على لقاح الأنفلونزا أعلى بين المصابين بأمراض الرئة، بينما كان الحصول على لقاح التهاب الكبد ب أعلى بين المصابين بأمراض القلب والأوعية الدموية، أو مرض السكري. كان الذكور، والمقيمون في المدن، والأسرة الأكثر ثراءً، وسنوات التعليم الأكثر، والظروف الطبية الحالية، والسلوكيات المستقرة، من العوامل الهامة للتنبؤ بالحصول على اللقاح.

**الاستنتاج:**

هناك حاجة إلى سياسات وبرامج مستهدفة لتحسين تغطية التطعيم المنخفضة بين كبار السن في الهند، وخاصة بين من يعانون من أمراض مزمنة. يمكن لمزيد من الأبحاث التحقق من الحصول على اللقاح، والتردد في الحصول على اللقاح، والمعلومات المتعلقة باللقاح، وقنوات الاتصال لكبار السن، ومقدمي الرعاية الصحية.

## 摘要

**目的:**

评估印度老年人疫苗接种覆盖率并探索预测因素。

**方法:**

我们采用了印度国家老龄化纵向研究的数据，该研究源自 2017-2018 开展的全国性家庭调查。根据受访者的自填报告，我们计算了 64,714 名 45 岁或以上的印度成年人中，接种流感疫苗、肺炎球菌疫苗、伤寒疫苗以及乙肝疫苗的人口权重估计值。我们采用多变量二元逻辑回归分析法来核验疫苗接种的社会人口和健康相关的预测因素。

**结果:**

所研究的每种疫苗接种覆盖率均低于 2%。报告曾经接种流感疫苗的受访者的百分比预计为 1.5%（95% 置信区间，CI：1.4–1.6），接种肺炎疫苗的为 0.6% (95% CI: 0.6-0.7)，接种伤寒疫苗的为 1.9% (95% CI: 1.8-2.0)，接种乙肝疫苗的为 1.9% (95% CI: 1.8–2.0)。患有心血管疾病、糖尿病或肺病的受访者的疫苗接种率高于没有任何这些疾病的受访者。患有肺病的受访者的流感疫苗接种率更高，患有心血管疾病或糖尿病的受访者的乙肝疫苗接种率更高。男性、城市居民、较富裕的家庭、受教育年限更长、现存医疗疾病和久坐行为是疫苗接种的重要预测因素。

**结论:**

需要有针对性的政策和计划来改善印度老年人（尤其是慢性病患者）疫苗接种覆盖率较低的情况。进一步的研究可以重点关注老年人及其医疗护理人员的疫苗获取、疫苗犹豫以及与疫苗相关的信息和沟通渠道情况。

## Резюме

**Цель:**

Оценить распространенность и изучить предикторы охвата вакцинацией людей старшего возраста в Индии.

**Методы:**

Авторы использовали данные национального продольного исследования в области старения населения в Индии, национального обследования домохозяйств, проведенного в 2017–2018 гг. На основе самоотчетов интервьюируемых лиц авторы рассчитали средневзвешенные популяционные значения охвата вакцинацией против гриппа, пневмококка, брюшного тифа и гепатита B среди 64 714 взрослых индийцев в возрасте от 45 лет и старше. Авторы провели многомерный анализ бинарной логистической регрессии, чтобы изучить социально-демографические и связанные со здоровьем предикторы охвата вакцинацией.

**Результаты:**

Охват каждой из изученных прививок составил менее 2%. Предполагаемый процент респондентов, сообщивших о любой своей вакцинации, составил 1,5% (95%-й ДИ: 1,4–1,6) для гриппа, 0,6% (95%-й ДИ: 0,6–0,7) для пневмококковой инфекции, 1,9% (95%-й ДИ: 1,8–2,0) для брюшного тифа и 1,9% (95%-й ДИ: 1,8–2,0) для гепатита B. Охват вакцинацией был выше среди респондентов с сердечно-сосудистыми заболеваниями, диабетом или заболеваниями легких, чем среди тех, у кого не было ни одного из этих состояний. Охват вакцинацией против гриппа был выше среди лиц с заболеваниями легких, а охват вакцинацией против гепатита В был выше среди лиц с сердечно-сосудистыми заболеваниями или диабетом. Важными предикторами охвата вакцинацией являлись мужской пол, проживание в городской местности, более обеспеченное домохозяйство, более долгий срок обучения, имеющиеся заболевания и малоподвижный образ жизни.

**Вывод:**

Необходимы целенаправленная политика и программы для улучшения низкого охвата вакцинацией людей старшего возраста в Индии, особенно среди людей с хроническими заболеваниями. Дальнейшие исследования могли бы изучить доступ к вакцинам, нерешительность в отношении вакцин, а также каналы информации и связи, связанные с вакцинами, для людей старшего возраста и медицинских работников.

## Introduction

Both the Global Vaccine Action Plan endorsed at the 2012 World Health Assembly^1^ and the Immunization Agenda 2030 endorsed in 2021 proposed a life-course approach to immunization to fight vaccine-preventable diseases.^2^ The coronavirus disease 2019 (COVID-19) pandemic has also underlined the importance of robust vaccination systems for control of vaccine-preventable diseases among older adults. In 2009 the Association of Physicians in India drew attention to the substantial burden of morbidity, disability and mortality due to infectious diseases among older adults in the country.^3^ The association recommended that vaccination was the most beneficial and cost-effective way to prevent and control infectious diseases in adults. Identification of vulnerable groups and the development of vaccination strategies for older people are therefore needed.

As reported in the World Health Organization’s (WHO) 2012 *Global report for research on infectious diseases of poverty*, diseases such as pneumonia, malaria, respiratory diseases, tuberculosis and typhoid pose a significant burden on low- and middle-income countries.^4^ Studies have shown that influenza and pneumococcal disease cause serious complications, especially in older adults with chronic illnesses.^5^ According to India’s National Centre for Disease Control, there were nearly 115 630 cases of H1N1 seasonal influenza in India between 2010 and 2017, accounting for 8685 deaths.^6^ Pneumococcal disease is a significant cause of bacterial pneumonia.^7^ The United States Centers for Disease Control and Prevention reported that pneumococcal pneumonia, resulting in one death in 20 adults (5%), has a high mortality in older adults.^8^ Likewise, acute hepatitis B virus (HBV) infection can cause severe illness and death and can lead to chronic HBV infection or liver cancer.^9^^,^^10^ The burden of HBV in India falls in the intermediate endemicity zone (prevalence of 2–7%, an average of 4%), affecting about 50 million people. The prevalence of HBV infection is higher in adults with diabetes.^11^ Typhoid is also a major burden on India’s public health system. In 2017, India had 6.6 million typhoid cases, accounting for 66 439 deaths.^12^ In 2008, the incidence of typhoid (493.5 cases per 100 000 per year) in India was the highest worldwide, followed by Pakistan, Indonesia, China and Viet Nam.^13^ Together, these infections are the leading causes of morbidity in older adults, resulting in hospitalization and death.

Due to declining fertility and increasing life expectancy, India is seeing a steady increase in the proportion of older people in the population. According to the 2011 Indian census, the adult population aged 45 years and older was 222 797 316 (18.4%) of the Indian population of 1 210 854 977. This figure is expected to rise to 40% (655 million people) by 2050.^14^^,^^15^ Besides the demographic transition, epidemiological transition has shifted the overall burden of disease towards older adults. Although chronic noncommunicable diseases have emerged as a primary concern, infectious diseases among such a large older population still pose a significant challenge to the Indian public health system.^16^

Studies on vaccination coverage among older adults are limited and mostly come from high-income countries. Researchers have reported that in people 65 years or older, seasonal influenza vaccination was received by more than 75% (8 363 467) of 10 341 592 people in the United Kingdom of Great Britain and Northern Ireland, 65% of 5332 people in the United States of America and 70% of 789 people surveyed in Canada.^17^^–^^19^ In comparison, a study from China reported that only 7.4% of 5414 people aged 60 years or older had received influenza vaccination.^20^ Many sociodemographic and health factors influence vaccine uptake among older adults.^21^^,^^22^ A systematic review of data from 12 countries found that older adults who received pandemic A(H1N1) influenza vaccination were more likely to be of higher education status, to have comorbidities and to have belief in vaccine efficacy.^21^ Another study found that previous vaccination history and physician recommendations were associated with vaccine uptake in older adults.^22^

Here, we aimed to analyse the uptake of four vaccines recommended for adults (influenza, pneumococcal, typhoid and hepatitis B) and the factors associated with their uptake among older adults in India. Such studies are essential for planning and implementing strategies to improve vaccination coverage among older adults in this large and diverse country. In addition, by identifying the underlying predictors of vaccination, we may be able to target vulnerable groups for future programme interventions.

## Methods

### Data source

We used data from the first wave of the Longitudinal Ageing Study in India conducted during 2017–2018. The study is a nationally representative survey of India’s health, economic, and social determinants and consequences of ageing. The study comprised interviews with 72 250 adults aged 45 years and older, including their spouses irrespective of age, across 30 states and six union territories of India. The response rate to the survey was 87.3% (72 250 out of 82 650 people approached). The study used a multistage, stratified, area-probability cluster-sampling design to collect data from both rural and urban areas. The details of the sampling design, survey instruments, fieldwork, data collection and processing are available elsewhere.^23^ We analysed information collected from 64 714 adults aged 45 years or older.

Ethical approval was not required for the study as we used publicly available data from a longitudinal study that used standard procedures for data collection with ethically approved guidelines and informed consent from participants.

### Data collection

The dependent variables were ever uptake of influenza, pneumococcal, typhoid or hepatitis B vaccines. Interviewees in the Longitudinal Ageing Study were asked: “Have you ever received any immunizations for adults, such as the influenza vaccine, pneumococcal vaccine, hepatitis B vaccine, or typhoid vaccine?” All the dependent variables were binary, with the response categories as Yes or No.

We extracted data on the sociodemographic characteristics of respondents: age (45–59, 60–69, 70–79, ≥ 80 years); sex (male, female); marital status (currently married, widowed, other); length of schooling (none, up to 5 years, 5–9 years, ≥ 10 years); working status (currently working, not working); social group (scheduled caste or tribe, other backward class, other); religion (Hindu, Muslim, other); wealth quintiles (poorest, poorer, middle, richer, richest); urban or rural area; and geographical region of residence (north, central, east, north-east, west, south). The Longitudinal Ageing Study reports wealth quintiles estimated from total monthly household expenditure. Scheduled castes or tribes and other backward classes are the constitutionally recognized groups of disadvantaged and deprived communities in India, and the Other category consisted of those who do not belong to these categories. The Other religion category included Christian, Sikh, other religions and no religion.

We also extracted data on health-related factors, including hospitalization in the past 12 months (yes, no); type of health care facility (public, private); physical activity (every day, sometimes, none); currently smoking (yes, no); ever alcohol consumption (yes, no); and having chronic disease(s) (yes, no). We considered sport or vigorous activity as a physical activity. Chronic diseases included at least one of hypertension, diabetes, cancer, lung disease, heart disease and stroke.

### Data analysis

First, we estimated the prevalence of uptake of the four types of vaccination among the respondents. Then we examined the prevalence of uptake of each type of vaccination by the respondents’ sociodemographic and health-related characteristics. Finally, we estimated four separate multivariable binary logistic regressions to study the factors associated with vaccine uptake. The statistically significant difference between the estimates was set at 5% level of significance. We used the survey weights given in the Longitudinal Ageing Study data set to estimate the prevalence of vaccination uptake for population size, adjusting for the complex design of the study to generate nationally representative estimates. We carried out the analysis using Stata version 14.0 (Stata Corp., College Station, USA).

## Results

### Sample characteristics

Around half of the 64 714 respondents, 33 694 (52.1%) were in the age group 45–69 years and 74.4% (48 139/64 712) were currently married; 30 068 were men and 34 646 were women. Nearly half (47.0%; 30 415/64 712) had received no schooling and 36.5% (17 129/46 987) were currently working. About three quarters (74.4%; 46 426/62 416) of respondents belonged to scheduled tribes, scheduled castes or other backward classes. Most respondents lived in rural areas (64.9%; 41 994/64 714) and reported their religion as Hindu (73.5%; 47 491/64 580). More than two thirds (70.2%; 38 873/55 353) of the interviewees reported no physical activity. Most respondents reported that they did not smoke (86.3% 55 515/64 346) or consume alcohol (81.9%; 52 735/64 367). Nearly half of respondents (38.6%; 24 955/64 714) reported having at least one chronic disease (Table 1).

**Table 1:** Estimated prevalence of vaccination for four vaccine-preventable diseases among adults aged 45 years or older by sociodemographic and health-related characteristics, India, 2017–2018

Variable	Total no. of respondents, unweighted^a^		Weighted no. (%) of respondents vaccinated			
			Influenza vaccine	Pneumococcal vaccine	Typhoid vaccine	Hepatitis B vaccine
**Age group, years**						
45–59	33 694		685 (1.4)	280 (0.5)	814 (1.9)	1 065 (1.9)
60–69	18 725		370 (1.6)	172 (0.7)	447 (2.0)	533 (1.9)
70–79	8 973		192 (1.6)	95 (0.8)	202 (1.9)	256 (1.8)
≥ 80	3 322		68 (1.5)	33 (0.8)	55 (1.0)	79 (1.4)
**Sex**						
Male	30 068		634 (1.6)	284 (0.7)	728 (1.9)	996 (2.1)
Female	34 646		681 (1.4)	296 (0.6)	790 (1.9)	937 (1.7)
**Marital status**						
Currently married	48 139		945 (1.5)	444 (0.7)	1 164 (2.0)	1 521 (2.1)
Widowed	14 391		293 (1.4)	120 (0.6)	318 (1.6)	349 (1.3)
Other	2 182		77 (2.3)	16 (0.6)	36 (1.1)	63 (1.5)
**Years of schooling**						
None	30 415		569 (1.5)	231 (0.6)	627 (1.7)	597 (1.3)
< 5	7 409		157 (1.7)	53 (0.5)	120 (1.7)	176 (1.6)
5–9	14 676		283 (1.5)	132 (0.7)	362 (2.1)	476 (2.1)
≥ 10	12 212		306 (1.3)	164 (0.8)	409 (2.2)	684 (3.5)
**Working status**						
Not working	29 858		396 (1.8)	157 (0.6)	381 (1.6)	463 (1.5)
Currently working	17 129		551 (1.4)	213 (0.5)	627 (1.8)	837 (1.7)
**Social group**						
Scheduled caste or tribe	22 101		368 (1.6)	171 (0.6)	457 (1.9)	692 (1.7)
Other backward class	24 325		540 (1.7)	156 (0.5)	457 (1.5)	469 (1.3)
Other	15 990		352 (1.1)	230 (1.0)	579 (2.6)	721 (3.1)
**Religion**						
Hindu	47 491		982 (1.5)	402 (0.6)	1 017 (1.7)	1 152 (1.6)
Muslim	7 674		107 (1.0)	55 (0.4)	129 (1.5)	143 (1.6)
Other	9 415		221 (2.8)	121 (1.1)	370 (4.7)	632 (5.4)
**Wealth quintile**						
Poorest	12 801		132 (0.7)	38 (0.2)	123 (0.9)	144 (0.8)
Poorer	13 030		184 (1.1)	86 (0.4)	258 (1.7)	273 (1.5)
Middle	12 993		234 (1.3)	109 (0.6)	301 (1.9)	334 (1.7)
Richer	13 035		309 (1.7)	137 (0.9)	359 (2.2)	466 (2.3)
Richest	12 855		456 (2.9)	210 (1.2)	477 (2.8)	716 (3.3)
**Urban or rural area**						
Rural	41 994		742 (1.6)	307 (0.6)	922 (1.9)	1 131 (1.8)
Urban	22 720		573 (1.3)	273 (0.7)	596 (1.7)	802 (2.1)
**Geographical region **						
North	11 587		372 (2.6)	287 (1.7)	846 (5.9)	695 (4.4)
Central	8 777		74 (0.8)	75 (0.9)	106 (1.2)	164 (2.0)
East	11 482		59 (0.4)	64 (0.5)	114 (1.2)	241 (2.2)
North-east	8 483		73 (0.6)	62 (0.5)	137 (1.6)	527 (5.3)
West	8 807		192 (0.4)	21 (0.1)	123 (1.9)	153 (0.5)
South	15 578		545 (3.5)	71 (0.3)	192 (1.1)	153 (0.6)
**Hospitalization**						
No	38 575		900 (1.6)	392 (0.7)	1 067 (2.1)	1 236 (2.1)
Yes	4 409		137 (2.6)	53 (0.7)	186 (3.6)	195 (2.6)
**Type of health-care facility**						
Public	10 321		252 (1.8)	87 (0.6)	245 (1.7)	345 (1.9)
Private	23 863		557 (1.5)	227 (0.6)	757 (2.3)	821 (2.1)
**Physical activity**						
Everyday	15 474		248 (1.1)	121 (0.4)	307 (1.7)	332 (1.4)
Sometimes	1 006		159 (1.4)	77 (0.6)	243 (2.1)	292 (1.9)
None	38 873		904 (1.7)	379 (0.7)	957 (1.9)	1 296 (2.1)
**Smoking^b^**						
Currently smoking	8 831		145 (1.6)	93 (0.9)	213 (2.0)	217 (1.7)
Not currently smoking	55 515		1 165 (1.5)	482 (0.6)	1 293 (1.9)	1 698 (1.9)
**Alcohol consumption**						
Yes	11 632		187 (1.5)	115 (0.7)	273 (2.0)	379 (2.2)
No	52 735		1 124 (1.5)	462 (0.6)	1 236 (1.9)	1 541 (1.8)
**Chronic disease(s)^c^**						
No	39 759		596 (1.1)	285 (0.5)	698 (1.5)	971 (1.6)
Yes	24 955		719 (2.1)	295 (0.9)	820 (2.5)	962 (2.4)
**Overall**	64 714		1 315 (1.5)	580 (0.6)	1 518 (1.9)	1 933 (1.9)

^a^ Total sample sizes vary due to missing information from respondents for the some of the variables analysed.

^b^ Not currently smoking includes past smokers and those who never smoked.

^c^ Persons with chronic diseases included those who have diabetes, cardiovascular disease, lung disease or cancer.

### Vaccination uptake

Table 1 shows the population-weighted numbers and estimated percentages of respondents who reported ever receiving the studied vaccines. Uptake was less than 2% for each of the vaccines.

An estimated 1315 respondents had received influenza vaccination, an overall uptake of 1.5% (95% confidence interval, CI: 1.4–1.6). Influenza vaccine uptake was slightly higher among men and among those who were not currently married or widowed, were not working, were neither Hindu nor Muslim, were in the wealthiest quintile, had been hospitalized in the last 12 months or had at least one chronic illness. Influenza vaccination uptake was highest in the south region of India followed by the north region. 

Pneumococcal vaccination was received by only 580 people (0.6%; 95% CI: 0.6–0.7). The uptake of pneumococcal vaccine was higher with older age and more years of schooling. Pneumococcal vaccine uptake was also higher among respondents who were working, were not of scheduled tribes, scheduled castes or other backward classes, were wealthier, had a chronic illness, were from the north region and were neither Hindu nor Muslim.

Vaccination for typhoid or hepatitis B was received by 1518 and 1933 respondents, respectively, showing an uptake prevalence of 1.9% (95% CI: 1.8–2.0) for each vaccine. The uptake of typhoid and hepatitis B vaccines also show similar patterns across the studied variables, except for urban or rural area. The prevalence of typhoid and hepatitis B vaccination was higher among older adults who were currently married, had 10 or more years of schooling, did not belong to scheduled tribes, scheduled castes or other backward classes, were neither Hindu nor Muslim, were wealthier, were from the north region, had a history of hospitalization, used private health-care facilities and had at least one chronic disease.

Vaccine uptake was higher among respondents with cardiovascular disease, diabetes or lung disease than those without any of these conditions (Table 1; Table 2). For example, estimated influenza vaccine uptake was 2.4% (95% CI: 1.9–2.9) among those with lung disease compared with 1.1% (95% CI: 1.0–1.2) among those with no chronic illness. The percentage of older people vaccinated against hepatitis B was higher among those with cardiovascular disease (2.5%; 95% CI: 2.3–2.8), diabetes (2.3%; 95% CI: 2.0–2.7) or cancer (4.7%; 95% CI: 2.7–6.7), compared with those without any of these conditions (1.6%; 95% CI: 1.4–1.7). For each vaccination type, observed differences in vaccine uptake among respondents with cardiovascular disease, diabetes or lung disease were not statistically significant.

**Table 2:** Estimated prevalence of vaccination among adults aged 45 years or older by history of chronic disease, India, 2017–2018

Vaccine	Overall^a^ (*n* = 64 714)	Chronic disease				
		Cardiovascular disease (*n* = 19 810)	Diabetes (*n* = 8 325)	Lung disease (*n* = 3 672)	Cancer (*n* = 434)	None (*n* = 39 957)
**Influenza vaccine**						
Weighted no. of vaccinees	1 315	438	181	89	5	450
Vaccine uptake, % (95% CI)	1.5 (1.4–1.6)	2.2 (2.0–2.4)	2.2 (1.9–2.5)	2.4 (1.9–2.9)	1.1 (0.1–2.1)	1.1 (1.0–1.2)
**Pneumococcal vaccine**						
Weighted no. of vaccinees	580	183	73	38	8	193
Vaccine uptake, % (95% CI)	0.6 (0.6–0.7)	0.9 (0.8–1.1)	0.9 (0.7–1.1)	1.0 (0.7–1.4)	1.8 (0.5–3.0)	0.5 (0.4–0.6)
**Typhoid vaccine**						
Weighted no. of vaccinees	1 518	535	187	89	19	593
Vaccine uptake, % (95% CI)	1.9 (1.8–2.0)	2.7 (2.5–2.9)	2.3 (1.9–2.6)	2.4 (1.9–2.9)	4.4 (2.5–6.3)	1.5 (1.4–1.6)
**Hepatitis B vaccine**						
Weighted no. of vaccinees	1 933	502	195	69	20	619
Vaccine uptake, % (95% CI)	1.9 (1.8–2.0)	2.5 (2.3–2.8)	2.3 (2.0–2.7)	1.9 (1.4–2.3)	4.7 (2.7–6.7)	1.6 (1.4–1.7)

CI: confidence interval.

^a^ Prevalence of uptake of any of the four vaccines was 4.2% (95% CI: 4.1–4.4).

Note: Sample sizes represent total unweighted number of respondents.

### Multivariable logistic regression

In our multivariable logistic regression analysis, the age of the respondents was not associated with uptake of any of the four types of vaccination (Table 3). Women were significantly less likely than men to have had influenza vaccine (odds ratio, OR: 0.64; 95% CI: 0.46–0.88) or hepatitis B vaccine (OR: 0.63; 95% CI: 0.46–0.87). Working people were more likely than non-working people to have received pneumococcal vaccine (OR: 2.19; 95% CI: 1.11–4.33), typhoid vaccine (OR: 1.43; 95% CI: 1.08–1.91) or hepatitis B vaccine (OR: 1.49; 95% CI: 1.11–2.01). Respondents of other religions (neither Hindu nor Muslim) were more likely to have been vaccinated than those of Hindu religion (ranging from OR: 3.19; 95% CI: 2.42–4.20 for hepatitis vaccine to OR: 1.41; 95% CI: 1.00–1.99 for influenza vaccine). Compared with the poorest wealth quintile, respondents in the richest wealth quintile were more likely to have received one or more of the vaccinations. While those from richer wealth quintiles were more likely to have received pneumococcal and hepatitis B vaccinations, respondents from the middle wealth quintiles were more likely to have received pneumococcal and typhoid vaccines.

**Table 3:** Predictors of vaccine uptake among adults aged 45 years or older by sociodemographic and health-related characteristics, India, 2017–18

Variable	OR (95% CI) of vaccine uptake			
	Influenza vaccine	Pneumococcal vaccine	Typhoid vaccine	Hepatitis B vaccine
**Age group, years**				
45–59	1.00 (ref.)	1.00 (ref.)	1.00 (ref.)	1.00 (ref.)
60–69	1.05 (0.77–1.44)	1.77 (0.98–3.19)	1.01 (0.75–1.36)	1.18 (0.89–1.57)
70–79	0.98 (0.65–1.48)	2.14 (0.86–5.35)	0.93 (0.63–1.37)	1.27 (0.86–1.88)
≥ 80	0.55 (0.26–1.19)	1.70 (0.51–5.65)	0.50 (0.26–0.95)	0.76 (0.39–1.48)
**Sex**				
Male	1.00 (ref.)	1.00 (ref.)	1.00 (ref.)	1.00 (ref.)
Female	0.64 (0.46–0.88)	0.76 (0.44–1.33)	0.93 (0.70–1.23)	0.63 (0.46–0.87)
**Marital status**				
Currently married	1.00 (ref.)	1.00 (ref.)	1.00 (ref.)	1.00 (ref.)
Widowed	0.90 (0.62–1.30)	0.85 (0.40–1.79)	0.93 (0.67–1.30)	0.96 (0.66–1.39)
Other	1.94 (1.14–3.27)	2.03 (0.80–5.15)	1.00 (0.51–2.00)	0.85 (0.43–1.65)
**Years of schooling**				
None	1.00 (ref.)	1.00 (ref.)	1.00 (ref.)	1.00 (ref.)
< 5	1.16 (0.77–1.76)	0.87 (0.41–1.87)	0.94 (0.61–1.44)	1.12 (0.72–1.73)
5–9	1.03 (0.73–1.44)	0.90 (0.50–1.60)	1.02 (0.75–1.38)	1.16 (0.85–1.58)
≥ 10	0.75 (0.50–1.13)	1.26 (0.76–2.09)	1.33 (0.95–1.86)	1.94 (1.44–2.60)
**Working status**				
Not working	1.00 (ref.)	1.00 (ref.)	1.00 (ref.)	1.00 (ref.)
Currently working	1.34 (0.99–1.83)	2.19 (1.11–4.33)	1.43 (1.08–1.91)	1.49 (1.11–2.01)
**Social group**				
Scheduled caste or tribe	1.00 (ref.)	1.00 (ref.)	1.00 (ref.)	1.00 (ref.)
Other backward class	0.71 (0.52–0.97)	0.92 (0.54–1.56)	0.83 (0.61–1.12)	1.05 (0.77–1.43)
Other	0.70 (0.49–0.99)	1.30 (0.72–2.34)	1.02 (0.75–1.39)	1.54 (1.14–2.09)
**Religion**				
Hindu	1.00 (ref.)	1.00 (ref.)	1.00 (ref.)	1.00 (ref.)
Muslim	0.54 (0.27–1.08)	0.43 (0.21–0.92)	1.03 (0.66–1.61)	0.90 (0.59–1.35)
Other	1.41 (1.00–1.99)	1.73 (1.02–2.95)	1.97 (1.45–2.68)	3.19 (2.42–4.20)
**Wealth quintile**				
Poorest	1.00 (ref.)	1.00 (ref.)	1.00 (ref.)	1.00 (ref.)
Poorer	1.37 (0.85–2.20)	1.36 (0.54–3.43)	1.63 (1.01–2.63)	1.41 (0.77–2.60)
Middle	1.52 (0.96–2.42)	2.40 (0.99–5.81)	1.83 (1.17–2.89)	1.36 (0.74–2.51)
Richer	1.36 (0.85–2.17)	3.21 (1.35–7.65)	1.51 (0.96–2.39)	2.24 (1.22–4.09)
Richest	1.86 (1.18–2.93)	5.21 (2.25–12.10)	2.17 (1.37–3.44)	2.97 (1.62–5.46)
**Urban or rural area**				
Rural	1.00 (ref.)	1.00 (ref.)	1.00 (ref.)	1.00 (ref.)
Urban	0.89 (0.66–1.20)	1.36 (0.82–2.24)	0.88 (0.65–1.19)	1.49 (1.16–1.91)
**Geographical region**				
North	1.00 (ref.)	1.00 (ref.)	1.00 (ref.)	1.00 (ref.)
Central	0.07 (0.02–0.21)	0.15 (0.06–0.38)	0.15 (0.10–0.25)	0.34 (0.21–0.55)
East	0.15 (0.09–0.26)	0.61 (0.35–1.08)	0.30 (0.21–0.44)	0.87 (0.65–1.16)
North-east	0.12 (0.06–0.23)	0.51 (0.20–1.30)	0.29 (0.18–0.49)	1.48 (1.09–2.01)
West	0.22 (0.13–0.40)	0.25 (0.09–0.71)	0.32 (0.22–0.46)	0.15 (0.08–0.30)
South	1.68 (1.25–2.26)	0.34 (0.19–0.63)	0.31 (0.23–0.43)	0.18 (0.12–0.27)
**Hospitalization**				
No	1.00 (ref.)	1.00 (ref.)	1.00 (ref.)	1.00 (ref.)
Yes	0.92 (0.57–1.50)	1.05 (0.57–1.93)	2.01 (1.46–2.77)	1.46 (1.04–2.06)
**Type of health-care facility**				
Public	1.00 (ref.)	1.00 (ref.)	1.00 (ref.)	1.00 (ref.)
Private	1.18 (0.88–1.57)	0.94 (0.52–1.68)	1.64 (1.23–2.18)	1.10 (0.84–1.43)
**Physical activity**				
Everyday	1.00 (ref.)	1.00 (ref.)	1.00 (ref.)	1.00 (ref.)
Sometimes	1.34 (0.88–2.05)	0.89 (0.49–1.65)	0.86 (0.61–1.21)	0.93 (0.66–1.32)
None	1.95 (1.44–2.65)	1.43 (0.83–2.44)	0.99 (0.73–1.33)	1.22 (0.90–1.67)
**Smoking^a^**				
Currently smoking	1.00 (ref.)	1.00 (ref.)	1.00 (ref.)	1.00 (ref.)
Not currently smoking	1.22 (0.84–1.77)	0.65 (0.36–1.18)	1.01 (0.78–1.49)	1.20 (0.86–1.68)
**Alcohol consumption**				
Yes	1.00 (ref.)	1.00 (ref.)	1.00 (ref.)	1.00 (ref.)
No	1.76 (1.25–2.47)	0.80 (0.47–1.37)	0.99 (0.78–1.49)	0.94 (0.86–1.68)
**Chronic disease(s)^b^**				
No	1.00 (ref.)	1.00 (ref.)	1.00 (ref.)	1.00 (ref.)
Yes	1.38 (1.06–1.80)	1.22 (0.78–1.91)	1.24 (0.97–1.59)	1.02 (0.79–1.31)

CI: confidence interval; OR: odds ratio; ref.: reference group.

^a^ Not currently smoking includes past smokers and those who never smoked.

^b^ Persons with chronic diseases include those who have diabetes, cardiovascular disease, lung disease or cancer.

Hospitalization in the past 12 months was significantly associated with higher uptake of typhoid vaccine (OR: 2.01; 95% CI: 1.46–2.77) and hepatitis B vaccine (OR: 1.46; 95% CI: 1.04–2.06). Respondents using a private health facility in the past 12 months were more likely to have received typhoid vaccination compared with those who used a public health facility (OR: 1.64; 95% CI: 1.23–2.18). Having any chronic disease was associated with a higher uptake of influenza vaccine (OR: 1.38; 95% CI: 1.06–1.80). 

## Discussion 

The uptake of the studied vaccines by older adults in India was considerably lower than that of higher income countries.^24^ Vaccination uptake ranged from 0.6% for pneumococcal vaccine to 1.9% for typhoid and hepatitis B vaccines. A 2020 study from China estimated a low, but slightly higher, prevalence of influenza vaccination among older adults (1651 out of 74 484 respondents; 2.4%).^25^ The low uptake may be explained by a lack of awareness and knowledge about vaccination for infectious diseases in adults, as noted by studies in India, Saudi Arabia and the USA.^26^^–^^29^ The study conducted in India found that out of 149 patients, only 2% and 0.7% had received influenza and pneumococcus vaccination, respectively.^26^ In a study of 832 diabetic patients admitted to a university hospital in Saudi Arabia, less than 40% of patients thought they were at a high risk of acquiring an infectious disease.^29^

Vaccination uptake among older adults in India varied by sociodemographic and health characteristics. Male sex, urban residence, existing medical conditions, more years of schooling and not engaging in physical activities were significant predictors of vaccine uptake. These findings are consistent with previous studies conducted in China and the USA among older adult populations.^18^^,^^27^^,^^30^ Higher vaccination coverage in urban areas was also evident in recent studies conducted in China.^25^^,^^31^ The uptake of influenza and pneumococcal vaccines was higher among older adults having lung diseases than those with other morbidities. Older adults with cardiovascular diseases were more likely to have received typhoid and hepatitis B vaccinations. A history of hospitalization was strongly linked to uptake of typhoid and hepatitis B vaccinations in our study. This finding is consistent with studies showing that health-care providers play an important role in motivating older adults to take up vaccination.^32^^,^^33^

For each of the four vaccinations, older adults from the richest wealth quintile households were significantly more likely to report being vaccinated than their counterparts from the poorest wealth quintile households. This finding is consistent with studies conducted in India and Poland that reported poor economic status as the most common barrier to vaccine uptake by adults.^26^^,^^34^ This finding is important given that poor people are at a higher risk of vaccine-preventable diseases and are disproportionally vulnerable to the economic impact of these diseases. Furthermore, expenditure on vaccinations among older adults is mainly funded out-of-pocket.^35^ In low- and middle-income countries many factors can impede the delivery of life-course immunization beyond childhood. These factors include an absence of policies for promoting adult vaccination, competing health priorities, lack of financing for health, heterogeneous populations and a dearth of research-based evidence. Fortunately, the Indian government has acted towards a healthy ageing approach by including special provisions for older adults in the health and wellness centres being opened across the country.^36^

Studies conducted in China and the USA have found regional variations in vaccine uptake by adults.^25^^,^^27^ In our study, the prevalence of uptake of pneumococcal, typhoid and hepatitis B vaccinations were highest in the north region of India. A higher prevalence of infectious diseases in the north region may be associated with the higher vaccination coverage.^37^ On the other hand, higher influenza vaccination coverage in the south region of India may be confounded by the higher prevalence of diabetes in the region.^16^ We also found a high vaccination coverage of hepatitis B in the north-east states. This could be due to the high prevalence of hepatitis B in the tribal-dominated region.^38^

Vaccine-preventable death has been used in several studies to measure the efficacy of the vaccination among older adults, as measured by uptake of childhood vaccination. However, vaccine-preventable disability might be a better indicator to assess the effectiveness of vaccines among older adults.^39^ Increasing vaccine uptake among older adults requires better availability and access to vaccines and improved financing and monitoring of vaccination systems. Engaging civil society and a proper campaigning strategy for vaccination may help counteract vaccine hesitancy among the public. The COVID-19 pandemic has opened a window of opportunity to build a mechanism for promoting adult immunization and a system to deliver the vaccines. Also, a life-course approach may be adopted for the needs of different groups of people in different regions of a country. Moreover, public and private health insurance policies may include vaccination for older adults with and without comorbidities. The role of global institutions such as WHO is important for global guidance on integrating the healthy ageing and universal health coverage agendas into Immunization Agenda 2030 and empowering low- and middle-income countries to develop adult immunization programmes.^40^

This study has some limitations. Our analysis was based on respondents’ self-reports which may be subject to recall bias. Second, our analysis was based on cross-sectional data and hence causal relationships cannot be established. Third, we did not explore the reasons for low vaccine uptake, access to vaccines and vaccine hesitancy among older adults in India. While influenza vaccine is valid only for one year, hepatitis B vaccine should be given as a series of two, three or four doses, depending on the vaccine manufacturer. For typhoid vaccine, a booster dose is needed every 5 years for people who remain at risk. We could not include vaccination time periods, doses or booster doses in our analysis due to lack of these data in the Longitudinal Ageing Study in India.

The findings of our study call for targeted policies and programmes for improving the vaccination coverage among older adults in India. Enhancing immunity, especially in the elderly population, can be beneficial in overcoming respiratory infections, including COVID-19. Health care and palliative care services for older people could be strengthened with the identification of high-risk groups and provision of vaccines for adults with chronic illness in India’s health and wellness centres.^36^ Monitoring vaccination coverage and evaluating vaccine efficacy is needed to reduce hospitalizations and deaths among older adults in India. Future research could assess access to vaccines and vaccine hesitancy within the older population, as well as information and communication channels to older adults and their health-care providers. Such steps will ensure health equity among the older adult population and create a platform to manage the current COVID-19 and future pandemics of infectious diseases.
